# Reported race-associated differences in control and schizophrenia post-mortem brain transcriptomes implicate stress-related and neuroimmune pathways

**DOI:** 10.3389/fnmol.2024.1450664

**Published:** 2024-11-18

**Authors:** Shay Simmons, Keon Arbabi, Daniel Felsky, Michael Wainberg, Shreejoy J. Tripathy

**Affiliations:** ^1^The Krembil Centre for Neuroinformatics, Centre for Addiction and Mental Health, Toronto, ON, Canada; ^2^Temerty School of Medicine, Institute of Medical Science, University of Toronto, Toronto, ON, Canada; ^3^Department of Psychiatry, Temerty School of Medicine, University of Toronto, Toronto, ON, Canada; ^4^Dalla Lana School of Public Health, University of Toronto, Toronto, ON, Canada; ^5^Lunenfeld-Tanenbaum Research Institute, Mount Sinai Hospital, Toronto, ON, Canada; ^6^Department of Physiology, Temerty School of Medicine, University of Toronto, Toronto, ON, Canada

**Keywords:** computational psychiatry, neuroscience, health disparities, transcriptomics, gene expression, population health, health outcomes, social genomics

## Abstract

**Background:**

The molecular mechanisms underlying racial disparities in schizophrenia (SCZ) illness courses and outcomes are poorly understood. While these differences are thought to arise partly through stressful social gradients, little is known about how these differences are reflected in the brain, nor how they might underlie disparate psychiatric outcomes.

**Methods:**

To better understand the neuro-molecular correlates of social gradients, SCZ, and their overlap, we analyzed post-mortem dorsolateral prefrontal cortex (DLPFC) RNAseq data from two racially diverse cohorts in the CommonMind Consortium (235 reported Black and 546 White, 322 SCZ cases and 459 controls) using differential expression and gene set variation analyses.

**Results:**

We observed differences in brain gene expression that were consistent across cohorts and reported race. A combined mega-analysis identified 1,514 genes with differential expression (DE) between reported race groups after accounting for diagnosis and other covariates. Functional enrichment analyses identified upregulation of genes involved in stress and immune response, highlighting the potential role of environmental differences between reported race groups. In a race-by-diagnosis interaction analysis, no individual genes passed statistical significance. However, 109 gene sets showed statistically significant differences, implicating metabolic and immune pathways.

**Conclusion:**

Our results suggest molecular mechanisms uniquely perturbed across reported race groups and identify several candidate pathways associated with SCZ in a reported race-dependent manner. Our results underscore the importance of diverse cohort ascertainment to better capture population-level differences in SCZ pathogenesis.

## Introduction

Schizophrenia (SCZ) is a severe mental disorder affecting 1% of the world’s population, characterized by debilitating symptoms, including hallucinations, delusions, and disordered thinking (Javitt, 2010; Jia et al., 2010; Stępnicki et al., 2018). North American epidemiological studies have reported significant racial disparities in the prevalence of SCZ, with some studies noting a nearly three times higher incidence in Black relative to White populations (Bresnahan et al., 2007; Schwartz and Blankenship, 2014). In addition, Black individuals with SCZ typically experience earlier onset and more severe symptoms, including a greater degree of cognitive impairment (Schwartz et al., 2019) and earlier ages of death (Olfson et al., 2015). These differences suggest the possibility of different underlying pathophysiologies of SCZ that may differ between Black and White populations (Bishop et al., 2022).

Although the factors that cause SCZ are not fully understood, they are considered to result from an interplay between genetic predisposition and external environmental factors (Forsyth et al., 2013; Woolway et al., 2022). Some environmental factors, known as social determinants of health, substantively influence health outcomes (Braveman and Gottlieb, 2014) and include race-associated social stressors, such as racism, systemic poverty, and institutional adversities (Al Abo et al., 2022). The ubiquity of these stressors is evidenced by recent findings that the majority of Black Americans report encountering pervasive chronic social stressors, with many experiencing employment discrimination (57%), biased police interactions (60%), and directed racial slurs (51%) (Bleich et al., 2019). Furthermore, Black individuals with SCZ report experiencing more social stress and discrimination than White individuals with SCZ (Bommersbach et al., 2023). Exposure to these chronic stressors can worsen psychiatric outcomes through triggering sustained stress responses, contributing to allostatic overload (Berger and Sarnyai, 2015).

Despite the heterogeneity of SCZ neuropathophysiology, stress and immune pathway dysregulation are common endophenotypes of the diagnosis (Van Kesteren et al., 2017; Ermakov et al., 2021; Bishop et al., 2022). Thus, it has been hypothesized that social determinants of health, which disproportionately affect marginalized groups, could impact the brain in ways that contribute to varying psychiatric outcomes (Alnæs et al., 2019). However, there is a lack of understanding of how experiencing such stressors might manifest in the brain (McEwen and Gianaros, 2010). This knowledge gap is partly attributed to the underrepresentation of diverse groups in molecular-genomic databases and longstanding disparities in biomedicine (Jooma et al., 2019; Mitchell et al., 2022).

Postmortem analysis of the human brain at the transcriptomic level is a valuable tool for identifying molecular signatures associated with population-level differences, such as those associated with psychiatric illnesses (Mimmack et al., 2002; Ramaker et al., 2017; Gandal et al., 2018; Hoffman et al., 2022). Furthermore, this tool can prove especially useful when characterizing molecular underpinnings of SCZ, as it can identify gene expression patterns resulting from environmental factors (Juarez et al., 2014). Thus, brain transcriptomics has the potential to provide new insight into the complex interplay of environmental and genetic factors that lead to the development of psychiatric illnesses. Previous work has described significant and reproducible gene expression changes in SCZ (Yuan et al., 2019; Yang et al., 2020). However, while there have been efforts to understand how genetic ancestry is associated with postmortem brain transcriptomes in complex disorders like Alzheimer’s Disease (Felsky et al., 2023; Sariya et al., 2020), efforts to understand how differences in race-associated lived experiences might impact brain gene expression and the potential for developing SCZ has been lacking (McEwen and Gianaros, 2010).

**Figure 1:**
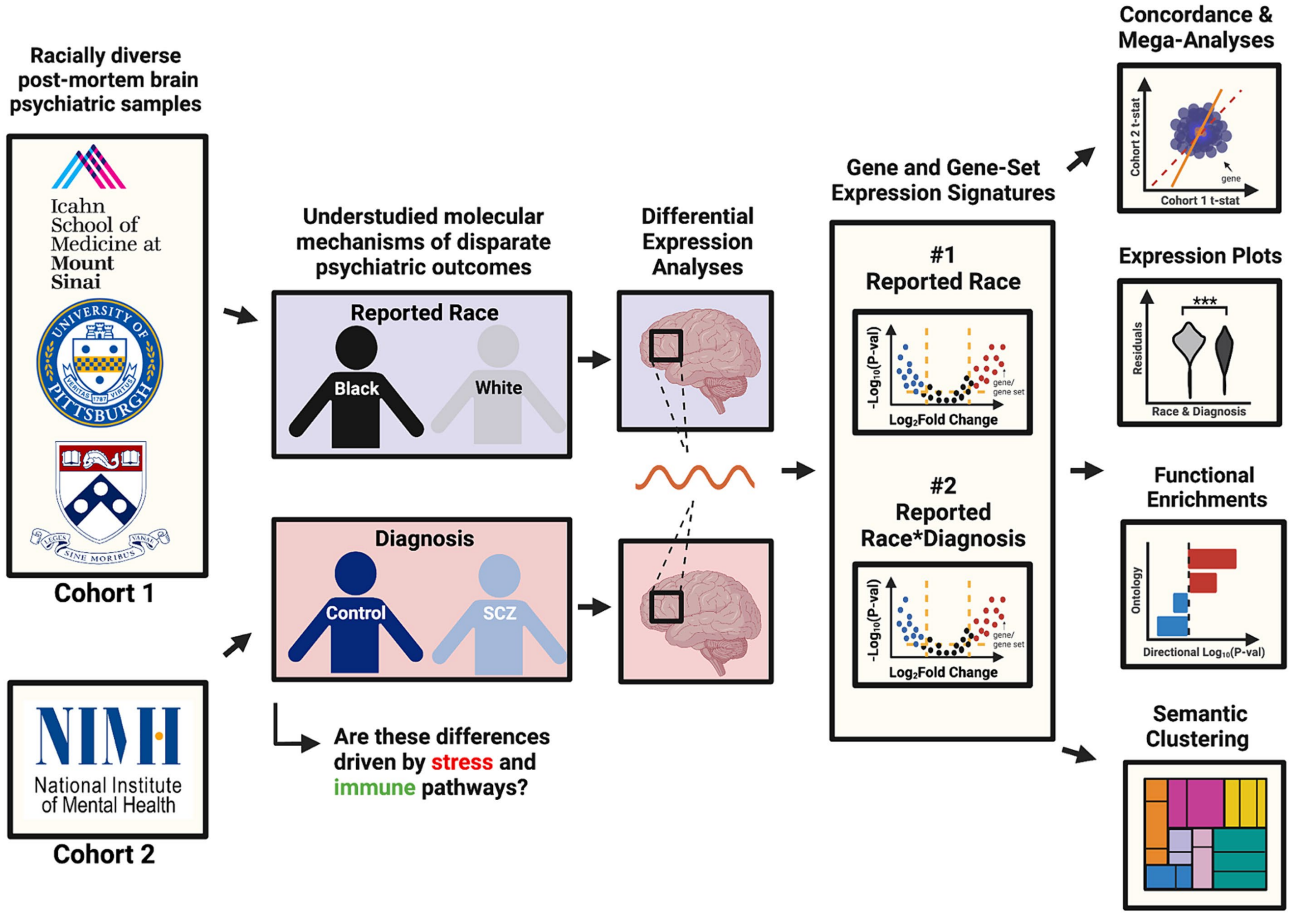
Visual abstract. This visual abstract outlines the study design and key goals related to the analysis of racial disparities in schizophrenia using post-mortem brain transcriptomic data from the CommonMind Consortium (CMC). The study integrates data from two major cohorts (MSSM-Penn-Pitt and NIMH-HBCC) and examines differential gene expression associated with reported race and its interaction with schizophrenia diagnosis.

Here, we aimed to better understand the mechanisms underlying racial disparities in schizophrenia (SCZ) by performing differential gene expression analyses in postmortem brain tissue RNAseq datasets from two large, racially diverse cohorts. As outlined in our visual abstract in Figure 1, our primary aims were to (1) identify genes and pathways that are consistently up- and down-regulated in brain samples across individuals reported as Black or White, reflecting exposure to social gradients associated with these groups; and (2) identify genes and pathways that are differentially expressed in SCZ in a reported race-specific manner using a reported race-by-diagnosis interaction model. We also aimed to assess how the reported race-associated effect overlaps with the *a priori* Conserved Transcriptional Response to Adversity (CTRA), an established gene expression signature that indexes responses to the exposure of chronic social stressors (Cole, 2009). We report that the gene expression signatures associated with reported race are reproducible across cohorts, strongly implicating cellular stress and immune pathways as predicted. Our study underscores the need for further analyses of racially diverse cohorts and statistical approaches that equitably consider lived experiences and social determinants of health.

## Methods

### Description of cohorts and sample filters

The experimental methods to generate the postmortem CommonMind Consortium (CMC) RNAseq dataset from the dorsolateral prefrontal cortex (DLPFC) are described in Hoffman et al. (2019). Data were downloaded from Synapse via synapse ID: syn2759792. As defined by Hoffman et al. (2022), the CMC comprises two cohorts collected across four institutions. The first cohort, MSSM-Penn-Pitt, consists of three institutions from the Mount Sinai School of Medicine Brain Bank, the University of Pennsylvania Brain Bank and the University of Pittsburgh Brain Bank. The second cohort, NIMH-HBCC, consists of one institution sampled from the National Institute of Mental Health’s Human Brain Collection Core.

**Figure 2:**
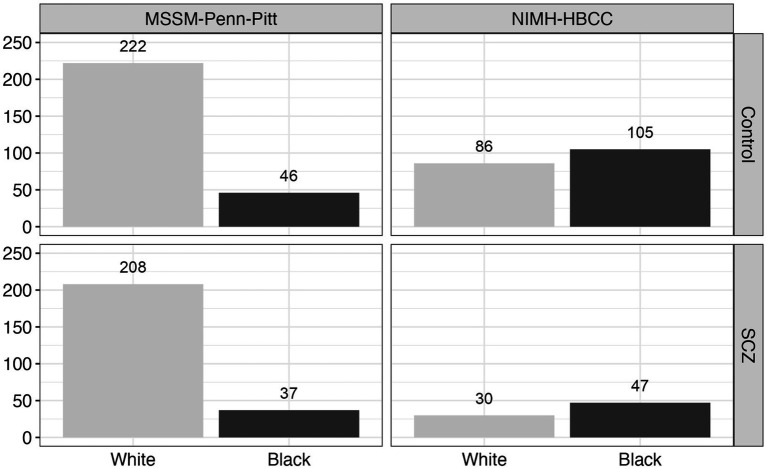
Reported race and neuropsychiatric composition across the CommonMind Consortium (CMC). This figure displays the distribution of reported race and neuropsychiatric diagnosis [Control vs. Schizophrenia (SCZ)] across two cohorts within the CommonMind Consortium (CMC): MSSM-Penn-Pitt and NIMH-HBCC. The bar plots depict the number of individuals reported as Black or White in each cohort and diagnosis group, highlighting the reported race composition and distribution of diagnoses within these cohorts.

We obtained reported race for each individual from metadata provided by the original authors and brain banks (Hoffman et al., 2019). Across each brain bank, participant race was reported by a next-of-kin or a lab technician. We note the possibility of discordance between this measure of reported race and how participants may self-identify had they been asked (see *Discussion*). Using available metadata provided by the CommonMind Consortium, we subset the total dataset to only include individuals with annotated chromosomal XX or XY sex, with control or SCZ psychiatric diagnosis, and Black or White reported race. We further filtered individuals without congruent sex and gender identity and removed RNAseq sample duplicates, leaving data from 781 unique individuals (Figure 2). In addition, we also made use of an available measure from the CommonMind Consortium, EV.1, that denotes a genotyping-based race variable (determined using genome-wide genotyping arrays) (Hoffman et al., 2019) to directly compare with our measure of reported-race. As expected, we observed a large degree of correspondence between reported race and genetically-inferred race (Supplementary Figure S1).

### Differential expression analyses and statistical covariates

We used processed RNAseq-based gene expression count data provided by the CommonMind Consortium and filtered subjects and samples as described above. We performed differential expression (DE) analyses to identify genes differentially expressed across samples reported as Black or White, control or SCZ diagnosis, and the reported race-by-diagnosis interaction. We identified differentially expressed genes using the ‘dream’ differential expression framework built on ‘limma-voom’ (Ritchie et al., 2015). To account for reported race, diagnosis, and the reported race-by-diagnosis interaction, we created a nested variable combining the reported race and diagnosis terms, then utilized contrasts implemented within ‘dream’ to disentangle effects.

Prior to performing DE analyses, we first performed a series of statistical tests to assess whether metadata factors differ between reported race groups within each institution in our study. Specifically, we compared factors such as age at death, cause of death, with a specific focus on cardiovascular-related deaths due to limited cause-of-death data in the CommonMind portal metadata (see Supplementary Figure S2). Additionally, we assessed brain pH, post-mortem interval (PMI), schizophrenia diagnosis, sex distribution, and RNA integrity number (RIN) to ensure that any observed gene expression differences were not influenced by these metadata variables.

The analysis (see Supplementary Table S1) broadly revealed that most metadata variables did not show significant differences between reported race groups across the institutions involved in our study. This consistency suggests that these factors–such as brain pH, post-mortem interval (PMI), schizophrenia diagnosis, sex distribution, and RNA integrity number (RIN)–are unlikely to introduce bias into our differential gene expression results. However, we note some significant differences (identified as *p* < 0.05) that warrant attention. Specifically, we identified that in the Penn institution, individuals reported as White had a significantly higher average age at death (75.85 ± 13.17 years, *n* = 79) compared to individuals reported as Black (66.08 ± 15.08 years, *n* = 13; *p* = 0.0138). Additionally, in the Pitt institution, a higher proportion of individuals reported as Black were diagnosed with schizophrenia (56%, *n* = 27) when compared to those reported as White (32%, *n* = 120; *p* = 0.0429). Interestingly, we observed the opposite pattern in the Penn cohort, where a higher proportion of individuals reported as White were diagnosed with schizophrenia (67%, *n* = 79) compared to individuals reported as Black (23%, *n* = 13; *p* = 0.0068). Furthermore, in the NIMH-HBCC cohort, individuals reported as Black had a significantly longer PMI (34.55 ± 18.86 h, *n* = 151) when compared to individuals reported as White (27.84 ± 14.06 h, *n* = 116; *p* = 0.0018) and were also older at death (43.36 ± 18.34 years, *n* = 151) compared to individuals reported as White (35.1 ± 20.57 years, *n* = 116; *p* = 0.000567).

When performing DE analyses, in light of some of the differences in metadata factors between reported race groups identified above, we modeled our statistical approach after Hoffman et al. (2022), who analyzed the same CommonMind-based datasets. Specifically, as in Hoffman et al. (2022), we included the following technical and biological covariates: RNA integrity number (RIN), intronic rate (IntronicR), intragenic rate (IntragenicR), intergenic rate (IntergenicR), ribosomal RNA rate (rRNA) and cellular fractions of oligodendrocyte (cellF1), GABAergic (cellF2) and glutamatergic cells (cellF3). When performing analyses using multiple institutions, we also included a covariate related to institutional brain bank. Also, following Hoffman et al. (2022), we performed three sets of DE analyses, one each for the two cohorts of MSSM-Penn-Pitt and NIMH-HBCC and a third mega-analysis that combined both cohorts.

Our statistical models were defined as:

Gene Expression ~ Reported Race + Dx + Reported Race:Dx + Covariates.

When visualizing the results of individual genes, we calculated gene expression residuals by regressing out covariates (as defined above) to visualize expression per gene.

### Gene set variation analysis and functional enrichments

We performed gene set variation analysis (GSVA) using the ‘GSVA’ package (Hänzelmann et al., 2013) to identify gene ontology (GO) sets that were differentially expressed with respect to our contrasts of interest. We employed this analysis, in part, to improve our statistical power to identify significant effects by pooling biologically related genes into gene sets. We followed the same statistical model and covariates as the mega-analysis gene-level differential expression analyses.

We procured gene sets from the 2021 ‘Human Biological Process’ sets from the data library provided by the ‘Enrichr’ tool (Chen et al., 2013; Kuleshov et al., 2016; Xie et al., 2021). Then, we added two additional gene sets comprising the *a priori* Controlled Transcriptional Response to Adversity (CTRA) gene set. We filtered only to include sets with 10–150 genes.

We used the ‘g:Profiler2’ package (Raudvere et al., 2019; Kolberg et al., 2020) to perform pathway enrichments against GO, KEGG, REACTOME, WikiPathways, miRTarBase, and TRANSFAC databases. We used the ‘rrvgo’ (Sayols, 2020) package to perform semantic clustering and visualize hierarchies of differentially expressed GO terms’ statistical significance and size. We only included the top 50 most significantly up-or down-regulated gene sets.

### Statistical significance and thresholding

We used ordinary least squares regression (OLS) using all genes’ t-statistics from the cohort-independent DE analyses to evaluate the concordance of differential expression signatures between cohorts. We used the Benjamini-Hochberg False Discovery Rate (FDR) (Benjamini and Hochberg, 1995) to account for multiple testing across genes, implemented using ‘limma-voom.’ We define genes and pathways as differentially expressed if they meet both criteria: an FDR < 0.05 and an absolute log_2_-fold change (LFC) threshold of 0.25.

## Results

### The brain transcriptomic signature of reported race is robust and enriched for stress-response and immune-related pathways

We used differential expression analyses to investigate the transcriptomic signature associated with reported race using post-mortem brain RNAseq datasets from the CommonMind Consortium (Hoffman et al., 2019). We used reported race as a proxy for social and environmental stressors associated with different racial groups, recognizing that health outcomes are largely shaped by systemic inequalities. Although we employed a binary classification for self-reported race, we acknowledge that race is a social construct influenced by a wide range of social and environmental factors (see *Discussion*). This measure of reported race was further highly concordant with genetic ancestry in these samples (Supplementary Figure S1). We performed this analysis using SCZ cases and controls, including covariates for age, sex, and other technical factors (see *Methods*) after careful consideration of how such metadata factors differ between reported race groups in each cohort (Supplementary Table S1).

**Figure 3:**
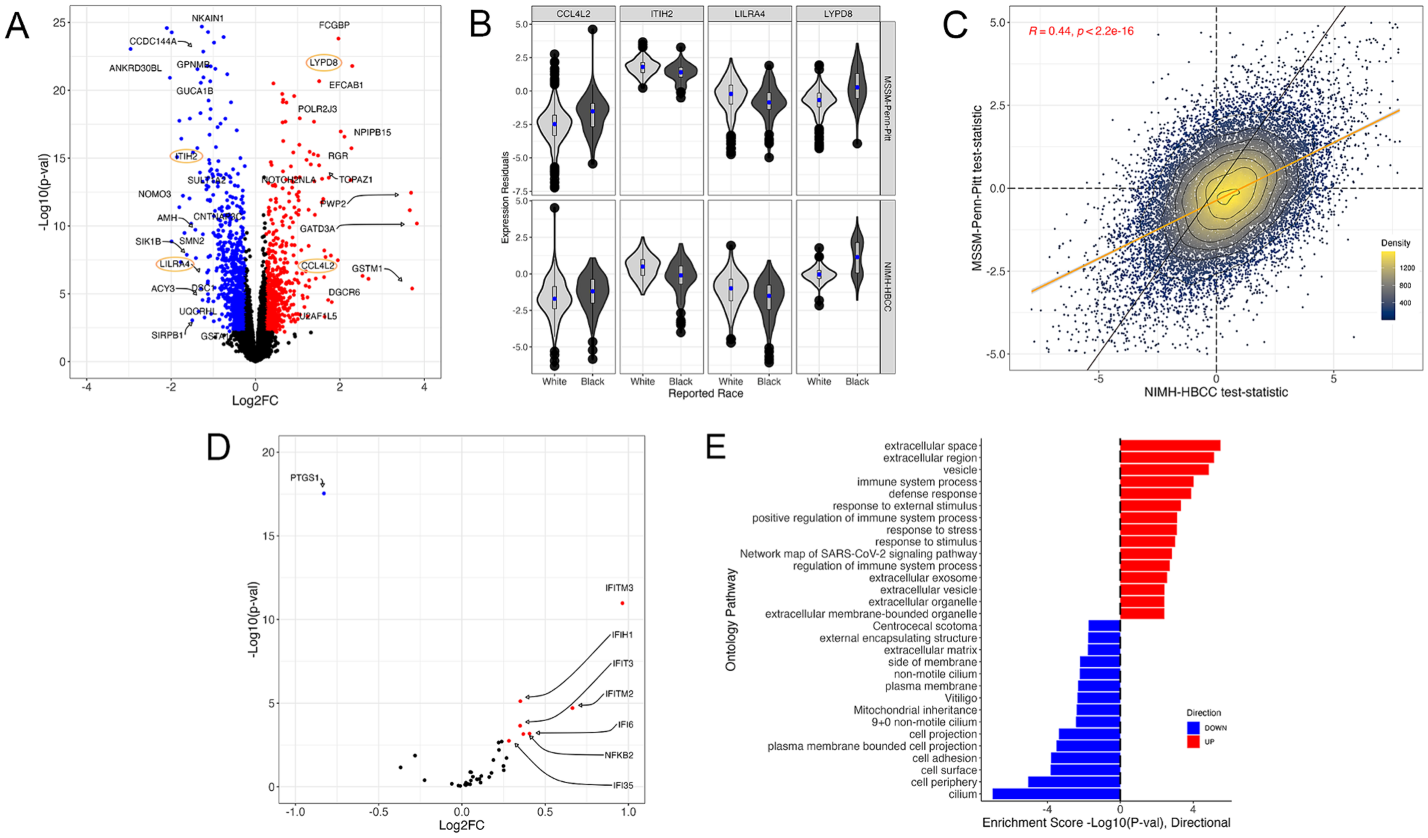
The gene expression signature of reported race. **(A)** Volcano plot illustrating genes differentially expressed across reported race groups using a mega-analysis that combines MSSM-Penn-Pitt and NIMH-HBCC cohorts. Blue (red) points indicate genes downregulated (upregulated) in individuals reported as Black relative to White with FDR < 5% and an absolute log_2_-fold change (LFC) of 0.25. Points circled in orange indicate genes plotted in **(B)**. **(B)** Residual expression of specific genes stratified by reported race and cohort. **(C)** Concordance analysis of reported-race-associated differential expression signatures between the MSSM-Penn-Pitt (y-axis) and NIMH-HBCC (x-axis) cohorts. Points indicate t-statistics from cohort-specific differential expression analyses and density lines, and the color of the points indicates local density. Differential expression analyses illustrate genes more highly expressed in individuals reported as Black (top, right) relative to White (bottom, left). The yellow line indicates the line of best fit, whereas the black line indicates the unity line. Inset R-value indicates Spearman’s correlation. **(D)** Volcano plot of the signature of reported race only illustrating genes in the *a priori* CTRA gene set. **(E)** Functional Gene Ontology (GO) enrichments for genes significantly up-(red) and down-regulated (blue) in individuals reported as Black relative to White.

Using the reported race measure, we identified 1,514 differentially expressed genes (DEGs) thresholded at a Benjamini-Hochberg FDR < 0.05 and an absolute log_2_-fold change (LFC) of 0.25 when combining both cohorts in a mega-analysis (*see Methods*) (Figure 3A, examples for individual genes shown in Figure 3B, full results in Supplementary Table S2). These DEGs included key genes involved in stress and immune biological processes, such as the C-C Motif Chemokine Ligand 4 Like 2 gene *CCL4L2* (LFC = 1.42; FDR = 3.54 × 10^−5^), Inter-Alpha-Trypsin Inhibitor Heavy Chain 2 gene *ITIH2* (LFC = −1.1; FDR = 1.03 × 10^−13^), Leukocyte Immunoglobulin Like Receptor A4 gene *LILRA4* (LFC = −1.12; FDR = 5.40 × 10^−5^) and LY6/PLAUR Domain Containing 8 gene *LYPD8* (LFC = 2.29; FDR = 4.64 × 10^−18^). Importantly, we observed that the gene expression signatures of reported race were highly concordant between the MSSM-Penn-Pitt and NIMH-HBCC cohorts when analyzed independently (Spearman’s rho = 0.44; *p* < 2.2 × 10^−16^, Figure 3C).

To understand the relationship between reported race and the well-established *a priori* Controlled Transcriptional Response to Adversity (CTRA) gene profile defined by Cole et al. (2015) (*see Methods*), we tested the overlap between 53 CTRA genes and those in our transcriptomic signature. This analysis revealed that reported race-associated DEGs overlapped with eight CTRA genes (hypergeometric *p*-value = 7.18 × 10^−5^, Figure 3D). Stratifying by direction of effect further uncovered that the genes upregulated in individuals reported as Black shared seven genes from the CTRA interferon subset (*IFI16, IFI35, IFI6, IFIH1, IFIT1, IFIT2, IFIT3, IFITM2, and IFITM3*) and one with the CTRA pro-inflammatory subset (*NFKB2*) (Figure 3D; Supplementary Figure S3A).

To investigate whether the top differentially expressed genes associated with reported race in our study overlap with known schizophrenia risk genes, we conducted a comparative analysis utilizing genome-wide association study (GWAS) summary statistics from Pardiñas et al. (2018). We first identified the genes most significantly differentially expressed between racial groups in our dataset. These genes were then cross-referenced with the schizophrenia-associated genes reported in the GWAS dataset (Supplementary Table S3). This analysis revealed an overlap of several genes, including GSDME (gasdermin E), PDE4B (phosphodiesterase 4B), SLC6A11 (solute carrier family 6 member 11), PRKCB (protein kinase C beta), and OPCML (opioid binding protein/cell adhesion molecule like).

Next, we performed a gene ontology (GO) analysis to contextualize the biological functions of DEGs associated with reported race (Figure 3E). We identified numerous GO terms, with many of the most significantly up-regulated terms relating to stress-response and immune pathways, including *immune system process* (4th most significant term, *p* = 9.64 × 10^−5^) and *response to external stimulus* (6th most significant term, *p* = 4.74 × 10^−3^) (Figure 3E). Plotting the t-statistics of these ontologies’ gene constituents demonstrated concordant associations between MSSM-Penn-Pitt and NIMH-HBCC cohorts (*immune system process*, rho = 0.36 and *response to stimulus*, rho = 0.37, Supplementary Figure S3B). Alongside these terms, we note enrichment in additional stress and immune-mediated pathways, including *defense response* (*p* = 1.29 × 10^−4^), *response to stress* (*p* = 7.77 × 10^−4^)*, and positive regulation of immune system process* (*p* = 7.63 × 10^−4^) (Figure 3D). In contrast, our analyses revealed downregulation in mostly cell-structure-related pathways in individuals reported as Black, such as *cell adhesion* (*p* = 1.59 × 10^−4^) and *cell periphery* (*p* = 8.90 × 10^−6^).

### Interaction models reveal association of metabolic and immune pathways with schizophrenia in reported race-dependent manner

Given the numerous genes associated with the reported race measure, we aimed to explore if there are distinct transcriptomic signatures for schizophrenia (SCZ) across reported race groups. To address this, we employed a reported race-by-diagnosis interaction model, adjusting for age, sex, and other technical factors (see *Methods*). This approach more directly accounts for reported race when compared to previous SCZ studies that either did not consider or only adjusted for race as a covariate (Hoffman et al., 2022; Katsel et al., 2005; Leirer et al., 2019).

**Figure 4:**
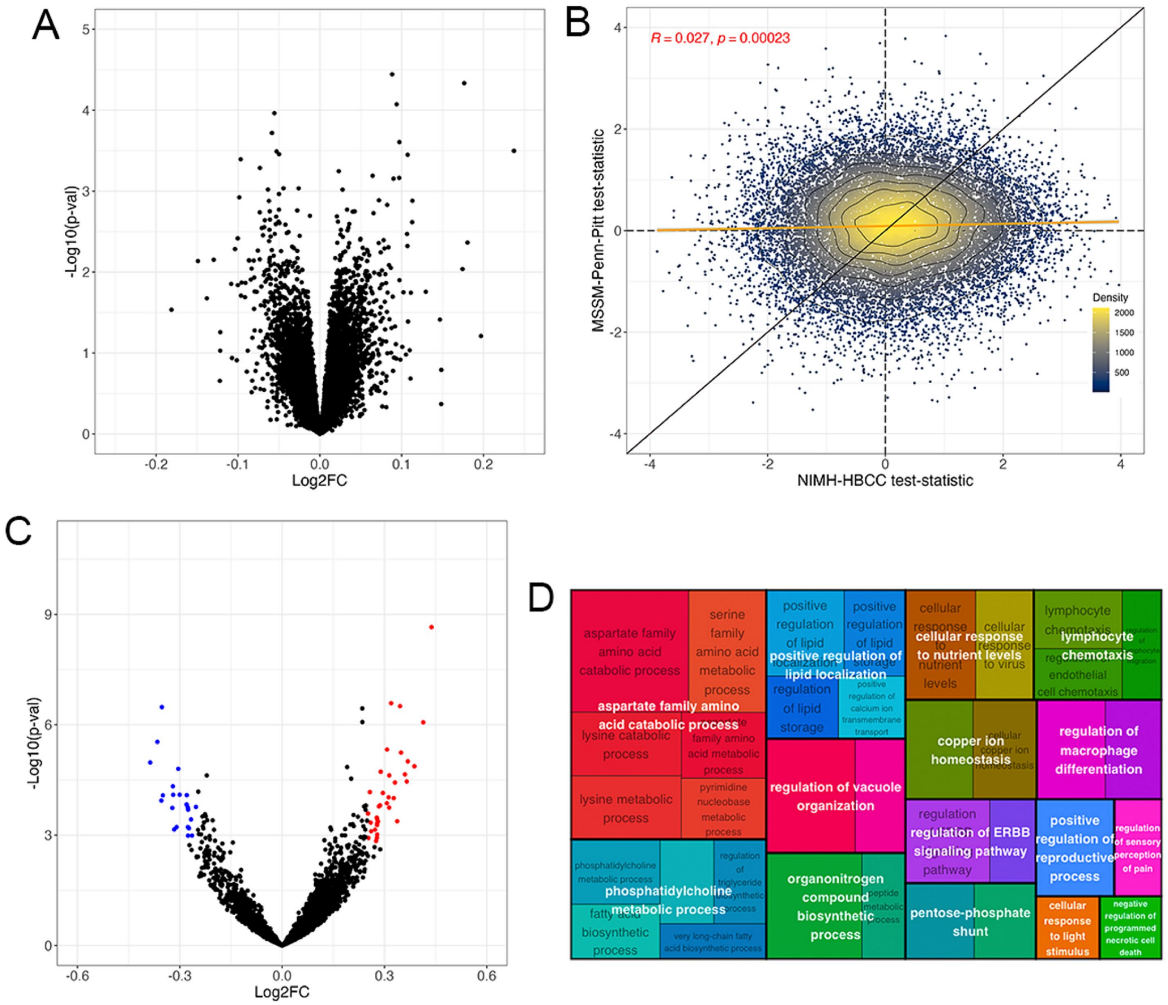
The gene expression signature of the reported race-by-diagnosis interaction. **(A)** Volcano plot of mega-analysis combining both cohorts indicating pathways enriched in individuals reported as Black relative to controls not seen in individuals reported as White (top, right) and in individuals reported as White relative to controls not seen in individuals reported as Black (top, left). **(B)** Differential expression analysis illustrates genes more highly expressed in SCZ cases reported as Black compared to controls not seen in individuals reported as White (top, right) and SCZ cases reported as White compared to controls not seen in individuals reported as Black (bottom, left) in the MSSM-Penn-Pitt (y-axis) and NIMH-HBCC (x-axis) cohorts. Points indicate t-statistics from cohort-specific differential expression analyses. The yellow line indicates the line of best fit, whereas the black line indicates a slope of one. The density lines and color of the points indicate local density. **(C)** Volcano plot of the GSVA revealing pathways enriched in individuals reported as Black relative to controls not seen in individuals reported as White (top, right) and individuals reported as White relative to controls not seen in individuals reported as Black (top, left). **(D)** Treemap plot of the enrichment analysis showing semantic clustering of top 50 terms expressed differently across controls reported as Black relative to SCZ cases not seen in individuals reported as White. Parent term (white text overlay) color is based on relatedness to the child term (black text underlay) and parent size is proportional to its statistical significance.

Differential expression analysis revealed no DEGs associated with the reported race-by-diagnosis term at Benjamini-Hochberg FDR < 5% (Figure 4A, full results in Supplementary Table S4). Accordingly, we observed a weak concordance between the MSSM-Penn-Pitt and NIMH-HBCC cohorts (rho = 0.027; *p* < 2.3 × 10^−4^, Figure 4B).

Having identified no significant differential expression at the single-gene level, we employed a GSVA to investigate interactions between reported race and SCZ diagnosis at the pathway level. Here, we identified 109 gene sets at an FDR < 5% and an absolute LFC threshold of 0.25 (Figure 4C, Supplementary Table S5). These included immune-related ontologies such as *negative regulation of leukocyte cell–cell adhesion* (LFC = −0.34; FDR = 9.13 × 10^−3^) and *neutrophil activation* (LFC = −0.25; FDR = 2.99 × 10^−4^). Contrarily we note positive changes in *cellular response to virus* (LFC = 0.36; FDR = 7.70 × 10^−3^) and, notably, *CTRA interferon* (LFC = 0.24, FDR = 4.04 × 10^−4^) gene sets (Figure 4C).

We then used semantic clustering to summarize the overall biological and relatedness across the top upregulated differentially expressed pathways. The most differentially expressed pathways belonged to parent GO terms involved in *aspartate family amino acid catabolic process,* but we note also observing immune-related pathways, such as *lymphocyte chemotaxis* (Figure 4D). A similar analysis of the downregulated ontologies revealed a relationship between *tetrapyrrole metabolic process striated muscle development* and *neutrophil activation* (Supplementary Figure S4). These results suggest that although the transcriptomic signature associated with the reported race-by-diagnosis interaction is only weakly concordant across cohorts at the single-gene level, gene set-based analyses indicate numerable significant gene sets at the pathway level.

## Discussion

Despite considerable research highlighting racial disparities in the clinical presentation and outcomes of schizophrenia (SCZ), studies investigating the underlying mechanisms remain limited. Our analysis of brain gene expression data from hundreds of individuals across four diverse brain banks represents one of the most extensive analysis of transcriptomic data aimed at characterizing gene expression differences associated with reported race and their interaction with SCZ.

By leveraging the CommonMind Consortium (CMC) dataset, we analyzed gene expression patterns associated with reported race in the brain, our analysis demonstrated robust reproducibility between the MSSM-Penn-Pitt and NIMH-HBCC cohorts. Consistent with our hypothesis, genes upregulated in individuals reported as Black were significantly enriched in pathways related to cellular stress and immune function, including *responses to stress* and *external stimuli*. This supports our hypothesis that reported race, used here as a proxy for social stress exposure, would be associated with differential expression in stress-response and immune-related gene expression, suggesting heightened social adversity. This aligns with prior research demonstrating that Black-identifying individuals exhibit dysregulated cellular stress and immune gene pathway expression in the context of blood leukocytes when exposed to increased social stressors (Thames et al., 2019).

In addition, genes upregulated in individuals reported as Black overlap with the well-established Conserved Transcriptional Response to Adversity (CTRA) gene set, specifically among the interferon gene-related subset of the CTRA. This finding aligns with previous research indicating that elevated CTRA gene expression is associated with higher levels of social stress (Knight et al., 2019; Lee et al., 2021). However, we note that the direction of such gene expression changes is somewhat inconsistent with the direction of changes predicted by the CTRA, which suggests that we would observe down-regulation of interferon related genes (Cole, 2009). Regardless, we recognize that the CTRA was initially established in the context of gene expression signatures derived from blood and immune cells, making it challenging to precisely translate such a signature to brain tissues. Furthermore, we note a precedence for discordant directions of effect between peripheral and brain tissues; for example, in early Alzheimer’s disease, amyloid levels increase in the brain but decrease in the cerebrospinal fluid (Hameed et al., 2020).

Our analysis of the reported race-by-SCZ interaction revealed no significant significantly associated DEGs at the single-gene level. These findings are similar to those of Hoffman et al., (2022), who also reported no genes significantly associated with a sex-by-diagnosis interaction effect using the same CMC dataset and analytical approach we use here. However, our gene-set-based GSVA analysis unveiled 109 significant differentially enriched gene sets associated with a reported race-by-diagnosis effect. These included pathways such as *negative regulation of leukocyte cell–cell adhesion* and *cellular response to stress*. These results confirm our hypothesis that the interaction of reported race and SCZ diagnosis would reveal the association of stress and immune gene sets.

A semantic clustering analysis on DE gene sets associated with the reported race-by-diagnosis interaction identified a trend for enrichment toward metabolic and immune processes, including multiple immune-related parent terms such as *lymphocyte chemotaxis* and *regulation of macrophage differentiation*. The most significant parent ontology, *aspartate family amino acid catabolic process*, is particularly notable, given the influence of amino acid metabolite levels on neural activity, offering a distinct understanding of chronic stress mechanisms (Ni et al., 2008).

A recent study by Benjamin et al. (2024) aimed to elucidate the impact of genetic ancestry on gene expression in postmortem brain tissue of neurotypical Black American individuals. Their study specifically focused on identifying differentially expressed genes (DEGs) associated with the proportion of African or European genetic ancestry. In contrast, our study takes a different approach by examining DEGs associated with reported race; a construct that encompasses environmental, cultural, and social factors. By focusing on reported race, our study considers the broader impact of sociocultural factors and their interaction with genetic predisposition, which is crucial for understanding racial disparities in mental health outcomes. Despite these important methodological differences, both studies converged on a key finding: the enrichment of immune-related pathways among the DEGs associated with race or genetic ancestry. The consistency of these findings across studies with different methodological approaches underscores the robustness of the observed immune pathway enrichment. The similar conclusions drawn between our study and that of Benjamin et al. provide mutual validation, increasing confidence in the biological significance of these immune-related gene expression changes.

Despite its contributions to understanding gene expression differences between reported race groups in SCZ our study has several noteworthy limitations. First, we used a measure of reported subject race provided by a next of kin or laboratory technician. However, this measure holds potential value over other measures, such as genetic ancestry, as it enables us to better assess the impact of stress originating from external sources. Unlike genetic ancestry, reported-race likely correlates better with individuals’ lived experiences, including social stressors. Second, we note that detailed sociodemographic information and environmental stressors experienced during participants’ lifetimes, including socioeconomic status, living quality, poverty level, and perceived discrimination, were unavailable or not collected from the subjects included in these studies. While we can reasonably expect that such factors would differ between reported groups studied here, we cannot be sure to what extent the reported race-associated differences we observed are due to differences in such environmental exposures or other factors. Furthermore, we acknowledge the unequal representation of Black individuals in the cohorts, with the MSSM-Penn-Pitt cohort having fewer individuals reported as Black compared to NIMH-HBCC, as well as the specificity of our sample, which was confined to individuals with a diagnosis of SCZ and controls.

Despite the molecular relatedness of SCZ to other psychoses, such as bipolar disorder (BD) (Cardno and Owen, 2014), both of which are known to share immune dysregulation (Torsvik et al., 2023), our findings may not be generalizable to other psychiatric or neurological conditions. Despite these limitations, our study provides important insights into how social gradients may impact the brain, potentially contributing to well-documented racial disparities in SCZ and other neuropsychiatric disorders.

In closing, our findings highlight a molecular signature linked to the reported race measure, reflecting differences in stress-response and immune pathways. This underscores the critical importance of diverse cohort ascertainment and modeling of socio-demographic stressors when considering molecular markers of SCZ. Our findings provide evidence linking reported race, used as a proxy of social stress, to differential expression in cellular stress response and immune-related genes in the brain and emphasize the potential contribution of environmental stressors to the distinct and divergent psychiatric outcomes observed among Black American populations. Moreover, our findings suggest the potential influence of environmental factors, including those related to Black-specific experiences like systemic discrimination. To deepen our understanding of these interactions, future research should prioritize comprehensive socio-demographic data collection alongside genetic data, allowing for a more fulsome capture of the complexity of lived experiences.

## Data Availability

The original contributions presented in the study are included in the article/Supplementary material, further inquiries can be directed to the corresponding author.
