# Supplementary material for: Reported race-associated differences in control and schizophrenia post-mortem brain transcriptomes implicate stress-related and neuroimmune pathways
Source: Front Mol Neurosci. 2024 Nov 18;17:1450664. doi: 10.3389/fnmol.2024.1450664 (PMC11609921; doi:10.3389/fnmol.2024.1450664)
Supplement: Supplementary file 1 [file Table_1.DOCX]

**Supplementary Figures**

**
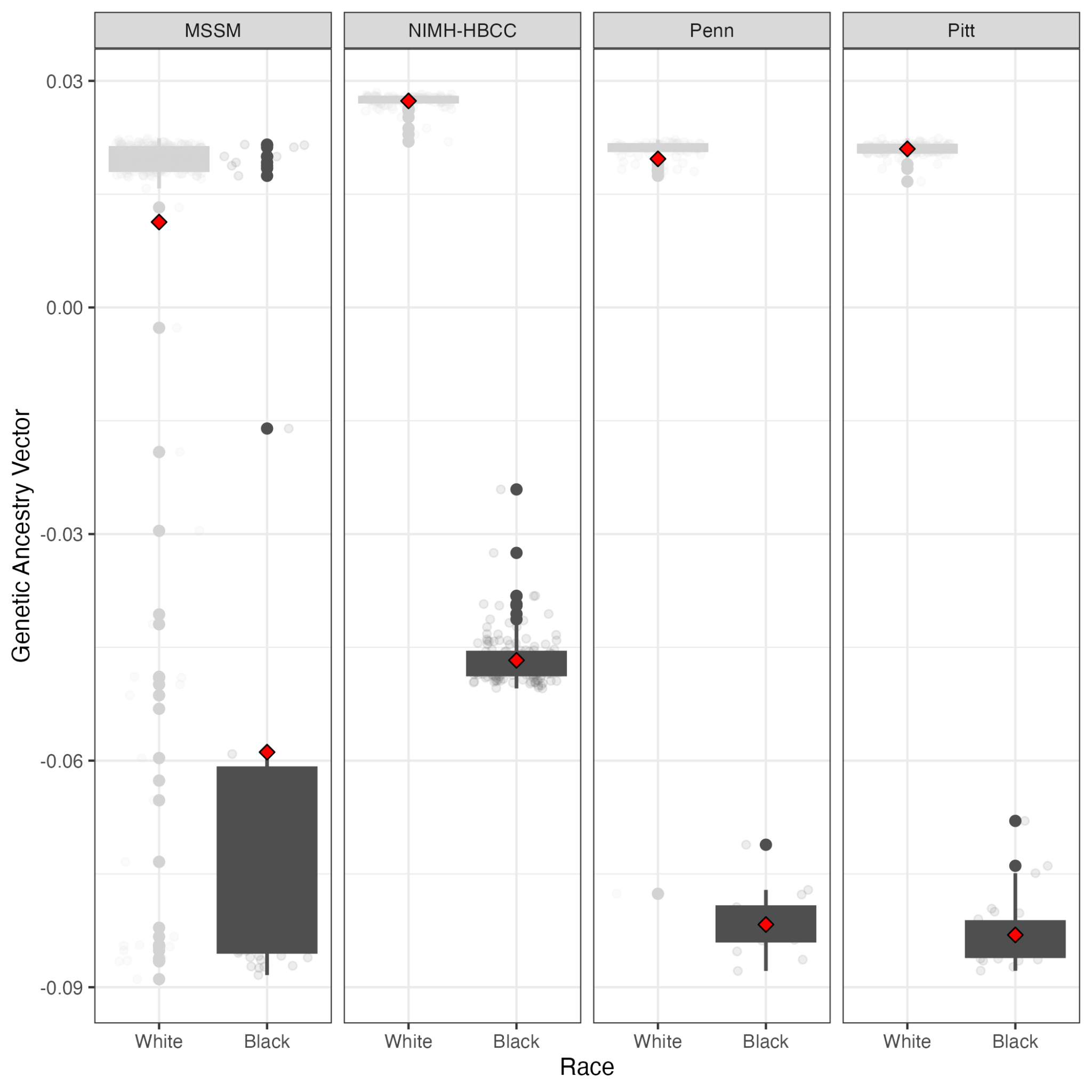
**

**Supplemental Figure 1. CMC Genetically Inferred Ancestry.** CommondMind Consortium genetically inferred ancestry measures across reported race and institutions based on measures available from [(Hoffman et al., 2019)](https://paperpile.com/c/lP2rYM/WHeG). We note that differences in the absolute magnitude of the y-axis, Genetic Ancestry Vector, between NIHM-HBCC and other institutions are based on different chips used for genotyping. Furthermore, we ensured that genetic ancestry direction vector directions were consistent between NIMH-HBCC and MSSM-Penn-Pitt cohorts.

**
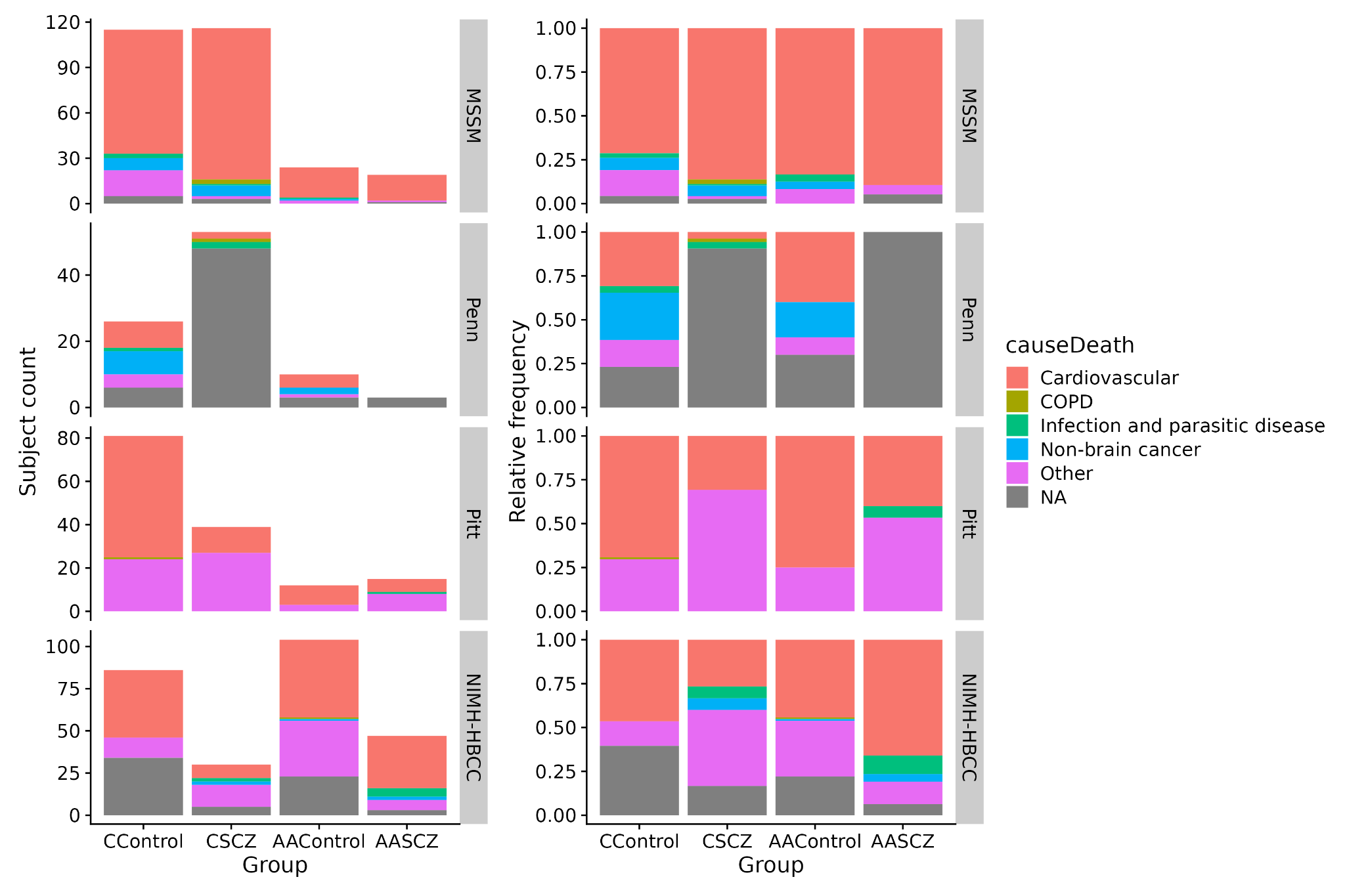
**

**Supplemental Figure 2. Distribution of Cause of Death Across Reported Race and Schizophrenia Diagnosis Groups by Institution.** This figure displays the distribution of cause of death across four different cohorts (MSSM, Penn, Pitt, and NIMH-HBCC) stratified by reported race (C: White, AA: Black) and schizophrenia diagnosis (Control vs. SCZ). The left panel shows the subject count for each cause of death category, while the right panel shows the relative frequency of each cause of death within each group. Causes of death are categorized into cardiovascular disease, COPD, infection and parasitic disease, non-brain cancer, and other causes, with an additional category for missing data (NA). The figure highlights the variability in cause of death across different racial and diagnostic groups within each institution, providing context for how these factors might influence postmortem brain gene expression analyses.


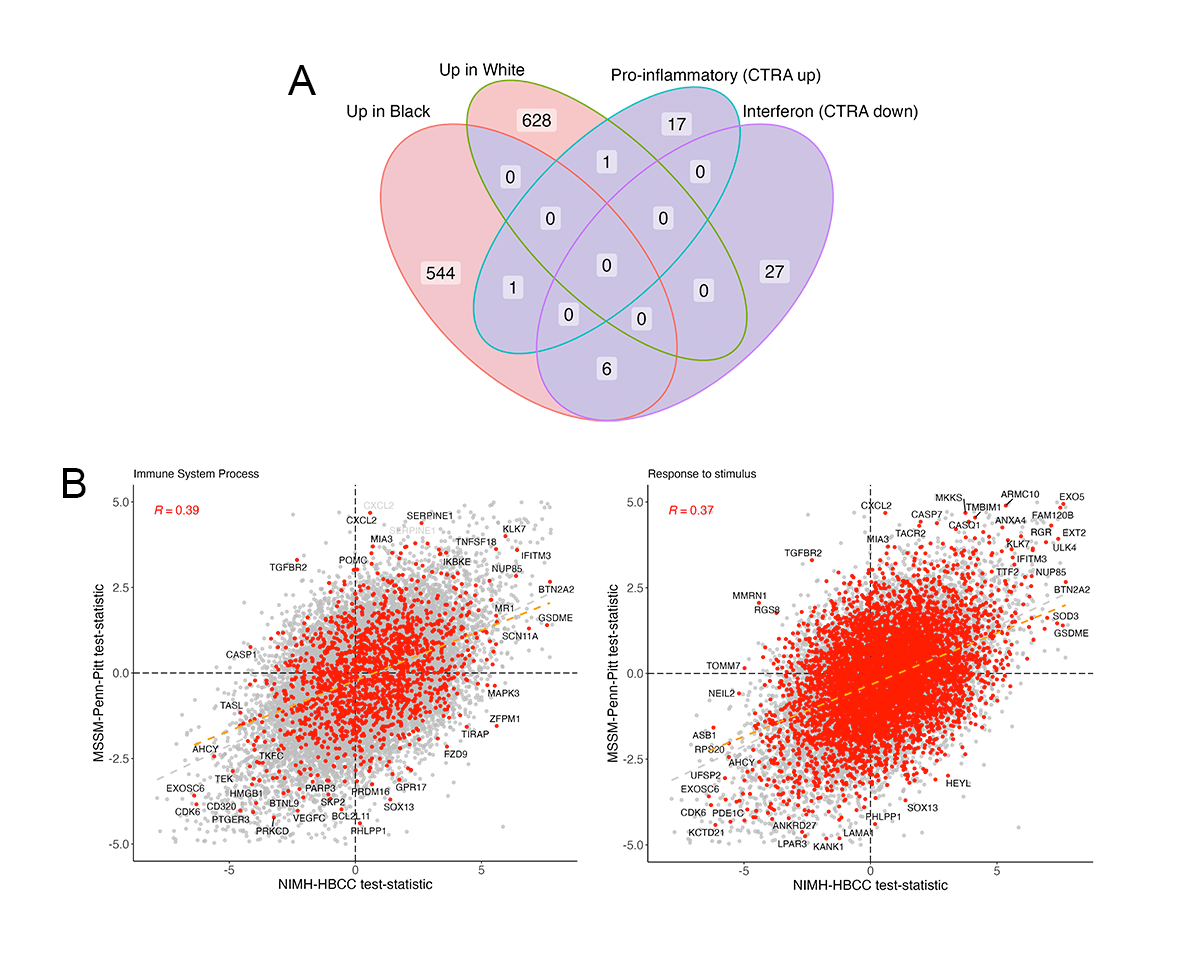


**Supplemental Figure 3. The Gene Expression Signature of Reported Race. (A)** Four-Way Venn comparing the gene expression signatures across reported race and the *a priori* CTRA, stratified by directionality and FDR < 5%. **(B)** Concordance of gene constituents in the *immune system process* and *response to stimulus* gene ontology terms across NIMH-HBCC and MSSM-Penn-Pitt.


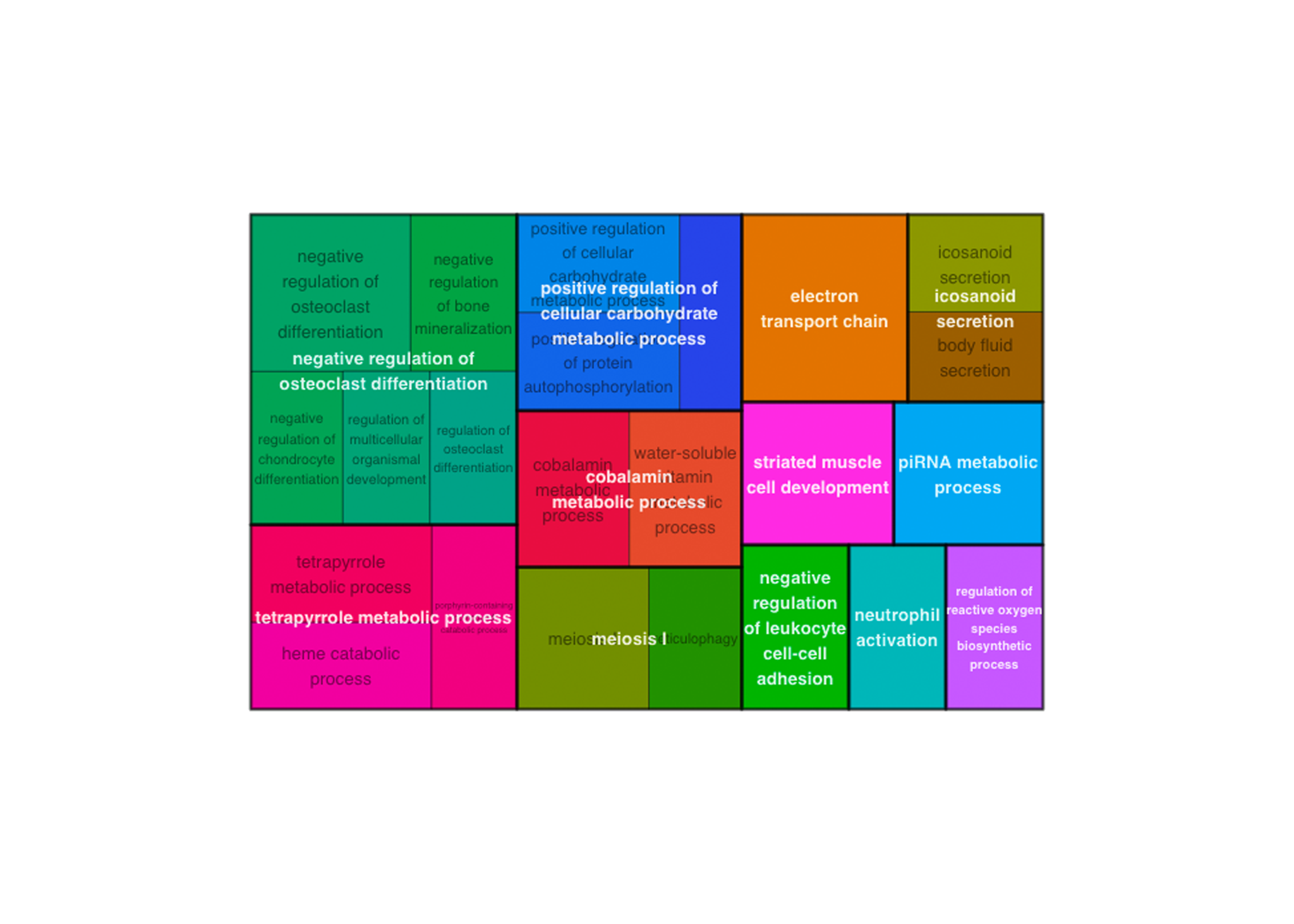


**Supplemental Figure 4. Semantic Clustering Analysis.** Treemap plot of the enrichment analysis showing semantic clustering of top 50 terms expressed differently across White controls relative to SCZ cases not seen in Black samples. Parent term (white text overlay) colour is based on relatedness to the child term (black text underlay), and parent size is proportional to its statistical significance.

**Supplementary tables**

**Supplemental Table 1. Comparison of Metadata Variables Across Racial Groups and Institutions in the CommonMind Consortium.** This table presents the comparison of key metadata variables between White and Black individuals across different institutions in the CommonMind Consortium dataset. The variables analyzed include age at death, cause of death (with a focus on cardiovascular-related deaths), brain pH, post-mortem interval (PMI), diagnosis of schizophrenia (Dx_SCZ), sex distribution (Sex_XY), and RNA integrity number (RIN). The goal of this analysis was to identify any significant differences (p < 0.05) between racial groups that might influence the differential gene expression findings. While most variables did not show significant differences across racial groups, a few noteworthy differences were identified in specific institutions, such as age at death, PMI, and the proportion of schizophrenia diagnoses.

**Supplemental Table 2. Title: Differential Expression Analysis of Reported Race .** This table presents the results of a differential expression analysis conducted to identify genes associated with reported race in a mega-analysis across the two main study cohorts. The analysis compares gene expression levels between reported Black and White individuals across the study cohort. Key metrics provided in the table include log fold change (logFC), average expression (AveExpr), t-statistic (t), p-value (P.Value), and adjusted p-value (adj.P.Val) for each gene.

**Supplemental Table 3. Intersection of Schizophrenia GWAS and Differential Expression Results from Reported Race Analysis.** This table presents the intersecting results of a genome-wide association study (GWAS) on schizophrenia with the differentially expressed genes (DEGs) identified from the reported race analysis in our study. The analysis aims to identify genes that are both significantly associated with schizophrenia risk, as indicated by the GWAS, and differentially expressed between reported Black and White individuals. The table includes key metrics such as gene symbols, log fold changes (logFC) from the differential expression analysis, p-values, adjusted p-values, and the corresponding GWAS information, including SNP loci (lead_variant) and distance to closest gene (distance).

**Supplemental Table 4. Title: Differential Expression Analysis of the interaction of Reported Race and Schizophrenia diagnosis.** This table presents the results of a differential expression analysis conducted to identify genes associated with the interaction of reported race and schizophrenia in a mega-analysis across the two main study cohorts. The analysis compares gene expression levels between reported Black and White individuals across the study cohort. Key metrics provided in the table include log fold change (logFC), average expression (AveExpr), t-statistic (t), p-value (P.Value), and adjusted p-value (adj.P.Val) for each gene.

**Supplemental Table 5. Reported Race-by-Diagnosis Interaction Term Results Using the GSVA.**

| **Pathway** | **log2FC** | **AveExpr** | **t-statistic** | **P.Value** | **adj.P.Val** | **B** |
| --- | --- | --- | --- | --- | --- | --- |
| **aspartate family amino acid catabolic process (GO:0009068)** | 0.4379102748 | -1.61E-17 | 6.053255272 | 2.22E-09 | 7.61E-06 | 10.8956728 |
| **detection of chemical stimulus involved in sensory perception of bitter taste (GO:0001580)** | 0.2349172057 | -3.04E-18 | 5.134821347 | 3.59E-07 | 0.0002455711813 | 6.219893982 |
| **electron transport chain (GO:0022900)** | -0.3524892039 | -2.14E-17 | -5.151002614 | 3.30E-07 | 0.0002455711813 | 6.296139211 |
| **organonitrogen compound biosynthetic process (GO:1901566)** | 0.3196770288 | -3.11E-17 | 5.198133305 | 2.59E-07 | 0.0002455711813 | 6.519485981 |
| **regulation of vacuole organization (GO:0044088)** | 0.3457688099 | 2.24E-17 | 5.162328697 | 3.11E-07 | 0.0002455711813 | 6.349639704 |
| **sensory perception of bitter taste (GO:0050913)** | 0.2351644619 | -4.66E-18 | 4.965646107 | 8.45E-07 | 0.0004210856942 | 5.436135012 |
| **serine family amino acid metabolic process (GO:0009069)** | 0.4136982515 | -2.18E-17 | 4.96188668 | 8.61E-07 | 0.0004210856942 | 5.418996498 |
| **negative regulation of osteoclast differentiation (GO:0045671)** | -0.3656434731 | 6.49E-18 | -4.712233793 | 2.91E-06 | 0.001245618688 | 4.308107796 |
| **cellular response to nutrient levels (GO:0031669)** | 0.3075739381 | 1.10E-17 | 4.609944475 | 4.72E-06 | 0.001795199875 | 3.868516195 |
| **positive regulation of reproductive process (GO:2000243)** | 0.3485530015 | 2.05E-17 | 4.569193878 | 5.71E-06 | 0.001953678522 | 3.695922821 |
| **regulation of ERBB signaling pathway (GO:1901184)** | 0.3684684567 | 1.01E-17 | 4.450290467 | 9.85E-06 | 0.003026466585 | 3.200602808 |
| **striated muscle cell development (GO:0055002)** | -0.3867231079 | 3.48E-18 | -4.433933128 | 1.06E-05 | 0.003026466585 | 3.133429442 |
| **detection of chemical stimulus involved in sensory perception of taste (GO:0050912)** | 0.1906767142 | -9.61E-18 | 4.370198862 | 1.41E-05 | 0.003224524813 | 2.87393343 |
| **lysine catabolic process (GO:0006554)** | 0.3877194727 | 1.10E-17 | 4.381250931 | 1.34E-05 | 0.003224524813 | 2.918676967 |
| **lysine metabolic process (GO:0006553)** | 0.3877194727 | 1.10E-17 | 4.381250931 | 1.34E-05 | 0.003224524813 | 2.918676967 |
| **piRNA metabolic process (GO:0034587)** | -0.3044752095 | 2.19E-17 | -4.346264322 | 1.57E-05 | 0.003363295687 | 2.777403662 |
| **regulation of macrophage differentiation (GO:0045649)** | 0.2890249008 | 5.36E-18 | 4.304278016 | 1.89E-05 | 0.003812172651 | 2.609285781 |
| **copper ion homeostasis (GO:0055070)** | 0.3140501951 | 2.89E-17 | 4.2519171 | 2.38E-05 | 0.004087346873 | 2.401800224 |
| **positive regulation of lipid localization (GO:1905954)** | 0.360102762 | 3.21E-18 | 4.267006884 | 2.23E-05 | 0.004087346873 | 2.461347389 |
| **regulation of cell communication (GO:0010646)** | -0.2208217531 | -1.00E-17 | -4.251328167 | 2.39E-05 | 0.004087346873 | 2.399480249 |
| **positive regulation of potassium ion transmembrane transporter activity (GO:1901018)** | 0.2025785822 | 2.44E-17 | 4.205570801 | 2.91E-05 | 0.004748235699 | 2.220164201 |
| **cellular response to virus (GO:0098586)** | 0.3651404996 | -1.00E-17 | 4.164200395 | 3.48E-05 | 0.005415534514 | 2.059631014 |
| **cellular copper ion homeostasis (GO:0006878)** | 0.3308755088 | -1.07E-17 | 4.149886149 | 3.70E-05 | 0.00550722143 | 2.004438445 |
| **meiosis I (GO:0007127)** | -0.3202845022 | 6.61E-18 | -4.093502838 | 4.70E-05 | 0.006705654797 | 1.788800664 |
| **positive regulation of interleukin-10 production (GO:0032733)** | -0.2457586866 | -4.40E-18 | -4.01410261 | 6.56E-05 | 0.008866523922 | 1.489913345 |
| **regulation of autophagosome assembly (GO:2000785)** | 0.2572004404 | 2.79E-17 | 4.007599862 | 6.73E-05 | 0.008866523922 | 1.465682949 |
| **phosphatidylcholine metabolic process (GO:0046470)** | 0.2955057415 | 2.09E-18 | 3.993668944 | 7.13E-05 | 0.009044915596 | 1.413900381 |
| **cobalamin metabolic process (GO:0009235)** | -0.3193786982 | -2.62E-18 | -3.966796199 | 7.97E-05 | 0.009131566887 | 1.314499364 |
| **negative regulation of leukocyte cell-cell adhesion (GO:1903038)** | -0.3495054741 | -2.47E-17 | -3.95779537 | 8.27E-05 | 0.009131566887 | 1.281349373 |
| **tetrapyrrole metabolic process (GO:0033013)** | -0.3003608586 | 1.28E-17 | -3.968047764 | 7.93E-05 | 0.009131566887 | 1.319114577 |
| **water-soluble vitamin metabolic process (GO:0006767)** | -0.2794661043 | 3.61E-18 | -3.961364251 | 8.15E-05 | 0.009131566887 | 1.29448491 |
| **aspartate family amino acid metabolic process (GO:0009066)** | 0.3120477803 | -1.35E-19 | 3.927487222 | 9.36E-05 | 0.01001282927 | 1.170255108 |
| **regulation of brown fat cell differentiation (GO:0090335)** | 0.3270434757 | -3.55E-17 | 3.914967569 | 9.85E-05 | 0.01021674455 | 1.124603276 |
| **negative regulation of bone mineralization (GO:0030502)** | -0.3540201101 | 1.79E-17 | -3.877689227 | 0.0001145310164 | 0.0115305785 | 0.9894987043 |
| **positive regulation of lipid storage (GO:0010884)** | 0.3071893403 | 1.45E-17 | 3.834886419 | 0.0001359782766 | 0.01329867545 | 0.83590206 |
| **positive regulation of cellular carbohydrate metabolic process (GO:0010676)** | -0.2799996745 | -1.72E-17 | -3.815378744 | 0.0001469632949 | 0.01397375996 | 0.7664423831 |
| **lymphocyte chemotaxis (GO:0048247)** | 0.2869003224 | -2.85E-17 | 3.801439229 | 0.0001553200176 | 0.01413985581 | 0.7170173494 |
| **microtubule anchoring (GO:0034453)** | 0.2404892297 | 1.97E-17 | 3.783500011 | 0.0001667349923 | 0.01413985581 | 0.6536664953 |
| **neutrophil activation (GO:0042119)** | -0.2525393794 | 1.21E-18 | -3.776485309 | 0.0001714099914 | 0.01413985581 | 0.6289729981 |
| **pentose-phosphate shunt (GO:0006098)** | 0.3136256778 | 1.64E-17 | 3.766941157 | 0.0001779694028 | 0.01413985581 | 0.5954459619 |
| **positive regulation of protein autophosphorylation (GO:0031954)** | -0.3218288705 | 3.17E-17 | -3.761580185 | 0.0001817568377 | 0.01413985581 | 0.5766495327 |
| **pyrimidine nucleobase metabolic process (GO:0006206)** | 0.2837937824 | -3.33E-17 | 3.785956232 | 0.0001651266302 | 0.01413985581 | 0.6623234146 |
| **rDNA heterochromatin assembly (GO:0000183)** | 0.2454505258 | -5.20E-18 | 3.798501012 | 0.0001571377465 | 0.01413985581 | 0.7066215602 |
| **regulation of reactive oxygen species biosynthetic process (GO:1903426)** | -0.2764891975 | 1.85E-17 | -3.768557623 | 0.0001768420681 | 0.01413985581 | 0.6011186021 |
| **heme catabolic process (GO:0042167)** | -0.2755560476 | -6.26E-18 | -3.735190272 | 0.0002015335539 | 0.01491619581 | 0.4844976313 |
| **phosphatidylethanolamine acyl-chain remodeling (GO:0036152)** | 0.2447355468 | -9.49E-18 | 3.731056662 | 0.0002048089989 | 0.01491619581 | 0.4701198731 |
| **porphyrin-containing compound catabolic process (GO:0006787)** | -0.2755560476 | -6.26E-18 | -3.735190272 | 0.0002015335539 | 0.01491619581 | 0.4844976313 |
| **fatty acid biosynthetic process (GO:0006633)** | 0.2516893406 | 2.02E-17 | 3.671345323 | 0.0002580776532 | 0.01840416264 | 0.2641385806 |
| **regulation of attachment of spindle microtubules to kinetochore (GO:0051988)** | -0.226133884 | -1.93E-17 | -3.659866649 | 0.0002697071794 | 0.01884097296 | 0.2249083858 |
| **positive regulation of protein localization to plasma membrane (GO:1903078)** | 0.2092696393 | 4.71E-19 | 3.64638314 | 0.0002839962217 | 0.01944238134 | 0.1789775743 |
| **negative regulation of lymphocyte activation (GO:0051250)** | -0.2239272534 | -1.29E-18 | -3.626414517 | 0.0003064687369 | 0.02042371226 | 0.111255853 |
| **regulation of mRNA metabolic process (GO:1903311)** | 0.222040482 | 3.56E-17 | 3.618165494 | 0.0003162304264 | 0.02042371226 | 0.08338473679 |
| **sulfate transport (GO:0008272)** | -0.233492269 | 3.29E-17 | -3.62230356 | 0.0003112976518 | 0.02042371226 | 0.09735843972 |
| **glucose 6-phosphate metabolic process (GO:0051156)** | 0.2776200356 | -2.15E-17 | 3.602882448 | 0.0003350912045 | 0.02124105913 | 0.03190943965 |
| **peptide metabolic process (GO:0006518)** | 0.2787759538 | 2.32E-17 | 3.579178976 | 0.000366441645 | 0.02280599547 | -0.04751100729 |
| **negative regulation of chondrocyte differentiation (GO:0032331)** | -0.2671839749 | 2.69E-17 | -3.573062309 | 0.00037496652 | 0.02291982854 | -0.06792327419 |
| **negative regulation of toll-like receptor signaling pathway (GO:0034122)** | -0.2193569864 | -1.96E-17 | -3.559696947 | 0.0003942445349 | 0.02316374527 | -0.1124082557 |
| **plasma membrane invagination (GO:0099024)** | -0.2038707228 | 9.32E-18 | -3.558204925 | 0.0003964532589 | 0.02316374527 | -0.1173642816 |
| **positive regulation of peptidyl-threonine phosphorylation (GO:0010800)** | 0.23400992 | -1.72E-17 | 3.551827506 | 0.0004060253334 | 0.02316374527 | -0.138525434 |
| **interferon** | 0.2487906152 | -2.16E-17 | 3.552673935 | 0.0004047425665 | 0.02316374527 | -0.1357189755 |
| **regulation of sensory perception of pain (GO:0051930)** | 0.3372177903 | 2.55E-18 | 3.544079097 | 0.0004179462157 | 0.02345294912 | -0.164186384 |
| **phosphatidylcholine biosynthetic process (GO:0006656)** | 0.2810816529 | -2.49E-18 | 3.539279907 | 0.0004254931563 | 0.0234913399 | -0.1800530532 |
| **membrane lipid catabolic process (GO:0046466)** | -0.2292414727 | 1.23E-17 | -3.52434133 | 0.0004498088294 | 0.02436318436 | -0.2293087195 |
| **regulation of endothelial cell chemotaxis (GO:2001026)** | 0.2595851586 | 1.79E-17 | 3.52094252 | 0.0004555196608 | 0.02436318436 | -0.2404872074 |
| **regulation of lipid storage (GO:0010883)** | 0.2788815562 | 2.46E-17 | 3.512372373 | 0.0004702220042 | 0.02476261416 | -0.2686276431 |
| **regulation of megakaryocyte differentiation (GO:0045652)** | 0.2068406019 | -1.61E-17 | 3.493629404 | 0.000503935577 | 0.02613593152 | -0.3299399302 |
| **ovarian follicle development (GO:0001541)** | 0.2000259786 | -8.73E-18 | 3.487820908 | 0.0005148334099 | 0.02630260839 | -0.3488763943 |
| **regulation of triglyceride biosynthetic process (GO:0010866)** | 0.2783469261 | -2.35E-17 | 3.477860891 | 0.0005340340928 | 0.02688233382 | -0.3812764372 |
| **antimicrobial humoral immune response mediated by antimicrobial peptide (GO:0061844)** | 0.2383304774 | -3.47E-17 | 3.460463459 | 0.0005691849323 | 0.02769931822 | -0.4376554676 |
| **L-serine metabolic process (GO:0006563)** | 0.2098596497 | 3.53E-17 | 3.462125024 | 0.0005657368138 | 0.02769931822 | -0.4322827285 |
| **negative regulation of myoblast differentiation (GO:0045662)** | -0.2326589922 | -3.08E-17 | -3.457901345 | 0.0005745403429 | 0.02769931822 | -0.4459352794 |
| **regulation of multicellular organismal development (GO:2000026)** | -0.309439734 | 4.73E-18 | -3.445407831 | 0.0006013348155 | 0.02828986307 | -0.4862247164 |
| **regulation of osteoclast differentiation (GO:0045670)** | -0.2761736475 | 3.10E-17 | -3.444503557 | 0.0006033187274 | 0.02828986307 | -0.4891353699 |
| **regulation of primary metabolic process (GO:0080090)** | 0.1417910117 | -2.29E-17 | 3.436596587 | 0.0006209278974 | 0.02872211071 | -0.5145545962 |
| **purine-containing compound salvage (GO:0043101)** | -0.2310026165 | -1.21E-17 | -3.421893811 | 0.0006549519895 | 0.02949869289 | -0.5616705498 |
| **reticulophagy (GO:0061709)** | -0.2731484269 | -1.73E-17 | -3.425418947 | 0.0006466395981 | 0.02949869289 | -0.550391856 |
| **copper ion transport (GO:0006825)** | 0.2438034483 | -1.37E-17 | 3.400693273 | 0.0007070804606 | 0.02968849261 | -0.6292645885 |
| **homologous chromosome pairing at meiosis (GO:0007129)** | -0.2464200538 | 1.78E-17 | -3.395711207 | 0.0007198787282 | 0.02968849261 | -0.6450899553 |
| **homologous chromosome segregation (GO:0045143)** | -0.199830334 | 9.64E-18 | -3.398166575 | 0.0007135445016 | 0.02968849261 | -0.6372933691 |
| **icosanoid secretion (GO:0032309)** | -0.3163835951 | -2.79E-17 | -3.403266732 | 0.0007005528662 | 0.02968849261 | -0.6210812793 |
| **mRNA destabilization (GO:0061157)** | 0.1846254438 | -1.78E-17 | 3.409783446 | 0.0006842732826 | 0.02968849261 | -0.6003320536 |
| **nucleic acid-templated transcription (GO:0097659)** | 0.1439506429 | 2.87E-17 | 3.399161062 | 0.0007109937727 | 0.02968849261 | -0.6341339993 |
| **regulation of lymphocyte migration (GO:2000401)** | 0.272318115 | -2.40E-19 | 3.398922399 | 0.0007116051367 | 0.02968849261 | -0.6348922856 |
| **positive regulation of calcium ion transmembrane transport (GO:1904427)** | 0.2607016264 | 2.57E-17 | 3.373873563 | 0.0007785620532 | 0.03172640367 | -0.7141912823 |
| **nucleoside diphosphate metabolic process (GO:0009132)** | -0.2440837842 | 4.18E-17 | -3.321892053 | 0.0009366134828 | 0.0377179759 | -0.8769386274 |
| **cellular response to light stimulus (GO:0071482)** | 0.2788225633 | -1.61E-17 | 3.314469309 | 0.0009614717363 | 0.03782893969 | -0.8999783088 |
| **sulfur compound transport (GO:0072348)** | -0.2376688281 | -3.80E-17 | -3.316982458 | 0.0009529876271 | 0.03782893969 | -0.8921832686 |
| **body fluid secretion (GO:0007589)** | -0.2757543705 | 7.21E-18 | -3.295026855 | 0.001029514713 | 0.0387255919 | -0.9600892859 |
| **positive regulation of protein deacetylation (GO:0090312)** | -0.2641692034 | 3.64E-18 | -3.29652272 | 0.001024125122 | 0.0387255919 | -0.9554766494 |
| **regulation of cell death (GO:0010941)** | 0.1846463164 | -6.78E-18 | 3.300352561 | 0.00101044525 | 0.0387255919 | -0.9436577224 |
| **tRNA 5'-end processing (GO:0099116)** | 0.1757003993 | 1.85E-17 | 3.301118119 | 0.001007731147 | 0.0387255919 | -0.9412936059 |
| **ceramide catabolic process (GO:0046514)** | -0.2129026891 | -2.62E-17 | -3.287995646 | 0.001055202267 | 0.03926040609 | -0.9817434417 |
| **alpha-amino acid catabolic process (GO:1901606)** | 0.2402266596 | 5.98E-18 | 3.27593404 | 0.001100655903 | 0.04051123822 | -1.018785232 |
| **dicarboxylic acid catabolic process (GO:0043649)** | 0.2304195718 | -2.83E-18 | 3.269376061 | 0.001126124199 | 0.04057603297 | -1.038869645 |
| **negative regulation of gene expression, epigenetic (GO:0045814)** | 0.2442284014 | -6.73E-18 | 3.269553495 | 0.001125428 | 0.04057603297 | -1.038326753 |
| **chemokine-mediated signaling pathway (GO:0070098)** | 0.2528993175 | 1.60E-17 | 3.253065837 | 0.001191851838 | 0.04183154823 | -1.088651737 |
| **negative regulation of programmed necrotic cell death (GO:0062099)** | 0.278011543 | 6.78E-18 | 3.256156317 | 0.001179131268 | 0.04183154823 | -1.079237535 |
| **regulation of cytosolic calcium ion concentration (GO:0051480)** | -0.2245866102 | -1.07E-17 | -3.251671799 | 0.001197631238 | 0.04183154823 | -1.092895404 |
| **cellular response to starvation (GO:0009267)** | 0.1557734677 | 9.64E-18 | 3.244554568 | 0.001227544276 | 0.04244327331 | -1.114533826 |
| **regulation of mRNA processing (GO:0050684)** | 0.2393694649 | 2.83E-17 | 3.24030069 | 0.001245751494 | 0.04264207365 | -1.127444844 |
| **positive regulation of DNA binding (GO:0043388)** | -0.2115879335 | 2.00E-18 | -3.224776731 | 0.001314336951 | 0.04454431074 | -1.174422259 |
| **negative regulation of DNA recombination at telomere (GO:0048239)** | 0.1238759068 | 2.35E-18 | 3.212731563 | 0.001369940452 | 0.04552724435 | -1.210721377 |
| **regulation of DNA recombination at telomere (GO:0072695)** | 0.1238759068 | 2.35E-18 | 3.212731563 | 0.001369940452 | 0.04552724435 | -1.210721377 |
| **positive regulation of lymphocyte differentiation (GO:0045621)** | -0.1967646999 | 1.44E-17 | -3.203628338 | 0.001413396838 | 0.04651978247 | -1.238067106 |
| **very long-chain fatty acid biosynthetic process (GO:0042761)** | 0.2755210507 | -6.71E-18 | 3.199670889 | 0.001432682886 | 0.04670546207 | -1.249931606 |
| **negative regulation of phosphorylation (GO:0042326)** | 0.1555113515 | 2.10E-17 | 3.190895468 | 0.001476318774 | 0.04767395436 | -1.276189604 |
| **inorganic anion transport (GO:0015698)** | -0.2352716012 | 1.40E-18 | -3.178457403 | 0.00154027185 | 0.04927430413 | -1.31328691 |
| **negative regulation of phosphate metabolic process (GO:0045936)** | 0.2162701448 | -1.67E-17 | 3.175163884 | 0.001557629188 | 0.04936819176 | -1.32308644 |
| **negative regulation of neuron death (GO:1901215)** | -0.1442335901 | -2.63E-17 | -3.169199511 | 0.001589522428 | 0.04991683734 | -1.340807675 |
